# Pathological Society

**Published:** 1897-01-09

**Authors:** 


### Medical Progress and Hospital Clinics.

*[The Editor will be glad to receive offers of co-operation and contributions from members of the profession. All letters should be addressed to* The Editor, at the Office, 28 & 29, Southampton Street, Strand, London, W.C.]

### PATHOLOGICAL SOCIETY.

Dr. A. A. Kanthack, associated with whom was Mr. T. W. Connell, read a paper on "Some Points in the Morphology of the Tetanus Bacillus." The most interesting of these had to do with the discovery of the flagellate phase of the bacillus. These flagella were easily stained, the nitrate of silver method giving the best results, and when so prepared the bacillus was seen to be completely surrounded by flagella, from twenty to thirty in number. It was, in fact, more freely provided with them than was the typhoid organism. Curiously, however, the bacillus moved very sluggishly. This, however, might be due to the fact that, as observed as a "hanging drop" preparation its condition was ærobic, whereas in its ordinary state it was an anærobic organism. After a time some of the flagella became much thickened, becoming what he called secondary flagella, while the rest of the thin primary ones dropped off, and ultimately only one single terminal flagellum was left, but when it approached the spore-bearing stage even this was lost.

Professor Crookshank thought some of the observations which had been recorded were not entirely novel, but in regard to the multiple flagella Dr. Kanthack had opened entirely new ground, and had his congratulations.

Dr. Douglas Drew showed specimens from a case of villous papilloma of the kidney, ureter, and bladder. The patient was fifty-six years old, and had been in University College Hospital under Mr. Barker. He had suffered for some months from severe pain in the left loin with hæmaturia, vomiting, and sweating. There was a tumour, soft and fluctuating. Nephrotomy was performed, but no stone was found, and the cavity was felt to be smooth. A month later, as the temperature was high and the kidney was not draining well, the wound was opened up, when a quantity of pus and flaky lymph was discharged. The patient, however, died, when there was found a villous carcinoma of the pelvis of the kidney with papillomatous growths in the ureter and in the bladder. Dr. Drew thought that the kidney had been the site of the original growth, which had at first been of the nature of a papilloma.

Mr. Lunn showed several specimens, the most interesting being a duct cancer, or columnar-celled carcinoma of the breast, removed from a man ninety-one years of age. An interesting discussion followed on the relation between so-called duct cancer, or, more accurately, columnar-celled carcinoma, and the ordinary scirrhous form, which was spoken of more definitely as spheroidal-celled carcinoma with free development of fibrous stroma.

